# A Perspective on Nanotechnology and COVID-19 Vaccine Research and Production in South Africa

**DOI:** 10.3390/v13102095

**Published:** 2021-10-18

**Authors:** Admire Dube, Samuel Egieyeh, Mohammed Balogun

**Affiliations:** 1 School of Pharmacy, University of the Western Cape, Bellville 7535, South Africa; segieyeh@uwc.ac.za; 2 Biopolymer Modification and Therapeutics Laboratory, Chemicals Cluster, Council for Scientific and Industrial Research, Brummeria, Pretoria 0001, South Africa

**Keywords:** COVID-19 and nanotechnology, nanomedicine in South Africa, bioinformatics and vaccine development, vaccine development in South Africa

## Abstract

Advances in nanotechnology have enabled the development of a new generation of vaccines, which are playing a critical role in the global control of the COVID-19 pandemic and the return to normalcy. Vaccine development has been conducted, by and large, by countries in the global north. South Africa, as a major emerging economy, has made extensive investments in nanotechnology and bioinformatics and has the expertise and resources in vaccine development and manufacturing. This has been built at a national level through decades of investment. In this perspective article, we provide a synopsis of the investments made in nanotechnology and highlight how these could support innovation, research, and development for vaccines for this disease. We also discuss the application of bioinformatics tools to support rapid and cost-effective vaccine development and make recommendations for future research and development in this area to support future health challenges.

## 1. Introduction

Within 18 months of a pandemic being declared, there were over 220 million confirmed COVID-19 infections and more than 4.6 million deaths globally [1]. Within the same time period, South Africa recorded over 2.8 million cases and over 85,000 deaths [2], the highest on the African continent. The causative organism of COVID-19 is the Severe Acute Respiratory Syndrome Coronavirus 2 (SARS-CoV-2). Coronaviruses are members of the subfamily *Coronavirinae* (family: *Coronaviridae*) that consists of four generations—alphacoronavirus, betacoronavirus, gammacoronavirus and deltacoronavirus—of which, only the betacoronaviruses SARS-CoV and MERS-CoV were regarded as being highly pathogenic prior to 2019 [3]. Acute respiratory distress syndrome as well as extrapulmonary manifestations have been the causes of death [4,5].

An unprecedented effort to develop vaccines to prevent COVID-19 infections and make them clinically available for mass immunizations globally started immediately once the causative organism was identified and its molecular signatures were elucidated in early 2020 [6,7]. The advanced drug delivery systems of nanoparticles have been applied to deliver nucleic acid-based vaccines to cellular and subcellular sites [8]. The research and development efforts for vaccines have been led, as they have traditionally been, largely by countries in the global north [9]. South Africa has emerged as an important global player with a membership in the community of five major emerging economies—which includes Brazil, Russia, India, and China—and is the only African member of the G20, which groups twenty countries that collectively account for 90% of the world’s economy. As an “emerging economy” country, it has invested tremendously in scientific research, development, and innovation (RDI) since the dawn of the 21st century. Here, we provide a synopsis of the investments made in nanotechnology by the South African government over the past 15 years as well as their RDI capabilities and highlight how these could support vaccine development for this disease. We also discuss the application of bioinformatics tools to support rapid and cost-effective vaccine development and make recommendations for future research and development in this area that will support preparedness for future health challenges. It is hoped that this perspective article will stimulate more collaborative innovation and product development aimed toward the local production of nanotechnology-based vaccines, therapeutics, and diagnostics for the country and the African continent.

## 2. Nanotechnology and Vaccines for COVID-19

Some of the vaccine formulations for COVID-19 that are currently used are nanotechnology-based. The mRNA in the Pfizer/BioNTech and Moderna vaccines are delivered in lipid nanoparticle formulations [6,8,10]. In this context, nanotechnology refers to the engineering of particles, which are in the nanoscale (1–1000 nm) and serve as systems to deliver a nucleic acid payload into cellular and subcellular sites in the body. Several reviews have been published, which highlight the utility of nanoparticles in protecting and enhancing the delivery of the mRNA or other antigens against SARS-CoV-2, and the reader is referred to these articles [11,12,13,14].

South Africa has yet to develop a COVID-19 vaccine, although researchers in the country also joined the global effort to discover an effective protective agent after the outbreak of the pandemic. The country has been recognized, along with Argentina, as a leader in molecular farming for the development of veterinary therapeutics and vaccines, given the significance of the local livestock industry [15]. The attenuation of a South African lumpy skin disease virus through the gene knockout of a virulent interleukin-10 gene homologue resulted in the demonstration of a vaccine that conferred protection against sheep pox and goat pox [16]. This capability can be conscripted in the development of vaccines against COVID-19.

The know-how to synthesize—and reproducibly manufacture at a large scale—various types of nanoparticles, including lipid nanoparticles, has been around for several decades, and several medicines based on nanotechnology are US Food and Drug Administration (FDA) approved. There are currently over 50 FDA-approved nanomedicines in clinical use [17,18]. Various types of nanoparticles are approved in these medicines, including liposomes, nanocrystals, metallic, polymeric, and lipid nanoparticles [18]. One of the early FDA approvals was in 1995 for a liposomal formulation of the anticancer drug doxorubicin [19]. Onpattro^®^ is an example of a lipid nanoparticle complex containing siRNA that was approved in 2018 [19]. In August 2021, the FDA granted full approval for the Pfizer mRNA lipid nanoparticle COVID-19 vaccine [20]. This endorsement is significant in many respects; not least is that the approved vaccine was developed less than a year after the emergence of the disease from a novel virus. It is also a strong signal of the potential benefits of investments in nanotechnology for modern health.

Nanotechnology has brought several therapeutics to patients over the years. Such medicines may not have made it to market due to unfavorable ‘drug-like’ properties, or have been repurposed and have had their safety profile and efficacy enhanced [21]. The application of nanotechnology in disease prevention and treatment is acknowledged globally [22,23]. In South Africa, extensive investment in the RDI landscape of nanotechnology formally began in 2005, with the launch of the National Nanotechnology Strategy [24]. Since this time, nanotechnology postgraduate training programs, research and development infrastructure, publications, patents, products, and companies have emanated [24,25]. National nanotechnology characterization centers include those at the Council for Scientific and Industrial Research (https://www.csirnano.co.za/; accessed on 1 October 2021) and the Department of Science and Innovation nanotechnology innovation centers for sensors and bio-labeling, located at Rhodes University and the University of the Western Cape, respectively [24].

## 3. Potential for Local Research and Development of Nanoparticle-Based COVID-19 Vaccines

A historically high burden of infectious diseases has meant that South African academic and RDI institutions, globally reputed for the quality of their outputs, have had decades of experience in offering cutting-edge solutions in this area. The country has the highest population of HIV-positive people, which has spurred research into nanoparticle-based antivirals. CAP256 is a potent anti-HIV antibody that was isolated from an HIV patient in South Africa in collaboration with international researchers [26]. This antibody has potential therapeutic and preventative properties. Further, CAPRISA 004, a topically applied anti-HIV microbicide was developed and underwent clinical testing in South Africa [27]. These experiences underscore the potential of South African scientists to respond to the need for new antivirals to fight SARS-CoV-2.

South African scientists discovered a new variant of SARS-CoV-2 (beta variant, also known as lineage B.1.351 with E484K and K417N mutations) that was subsequently responsible for the second wave of the disease in late 2020 to early 2021 [28]. The authors also demonstrated that plasma from patients who recovered from infection with the beta variant of SARS-CoV-2 could effectively neutralize the variant of the virus predominantly responsible for the initial wave of COVID-19 in 2020. However, the reverse was not true, as plasma from patients infected during the first wave was largely ineffective against the beta variant. Researchers in South Africa have also made contributions toward the understanding of the pathogenicity of coronavirus envelope proteins [29]. This makes the country the only one on the continent of Africa with the advanced technological capability and expertise to establish COVID-19 vaccine development and manufacture.

Nanoparticles for disease therapeutics have been investigated against infectious diseases, such as HIV/AIDS, malaria, tuberculosis, and cancer [30,31,32,33,34]. The application of ‘green nanotechnology’, synthesizing therapeutic nanoparticles using extracts of selected plants from the rich land and marine biodiversity of South Africa, has also been achieved. Plants, including *Sutherlandia frutescens*, *Cotyledon orbiculata,* and seaweed extracts of *Codium capitatum*, have been used to synthesize nanoparticles [35,36,37]. In July 2021, it was announced that Pfizer and BioNTech will—in collaboration with South Africa’s Biovac Institute, which already engages in vaccine manufacturing (https://www.biovac.co.za/; accessed on 1 October 2021)—begin production, in 2022, of around 100 million doses per year of their COVID-19 mRNA vaccine for the African continent [38]. Although this does not include the upstream synthesis of the mRNA, it does serve as an acknowledgment of the advancement and potential that exist in the country. A local pharmaceutical company, Aspen Pharmacare, is already in the downstream production of Johnson and Johnson’s viral vector COVID-19 vaccine [39]. The application of nanotechnology could also be extended to the delivery of protein-antigen-based vaccines. In the past, researchers at Stellenbosch University have reported the encapsulation of a bacteriocin, Plantaracin 423 (produced by *Lactobacillus plantarum* 423), in nanofibers, which was the first such report [40]. South Africa, therefore, has a great opportunity to innovate in this space and engage in the research, development, and manufacture of its own types of nanoparticles, mRNA, or protein-antigen vaccines for COVID-19. Such technology could also be applied to other diseases endemic in the country.

## 4. Bioinformatics Approaches to Optimize Research and Development

Bioinformatics pipelines are critical for predicting possible biomolecules that contribute to the understanding of infectious disease mechanisms, therapy, and prevention. Bioinformatics tools and approaches for analyzing biological data generated by genomes, transcriptomics, proteomics, and structural omics are gaining traction for the design of vaccines. These bioinformatics pipelines have been used in South African institutions (e.g., the South African National Bioinformatics Institute) to analyze the sequence of the SARS-CoV-2 genome from the South African population in order to provide a genetic “fingerprint” that can improve understanding and contain the spread of COVID-19 [41,42]. Recently, South African researchers could implement bioinformatics strategies of reverse vaccinology and immuno-informatics in vaccine design and development on a new big data platform for data-intensive research in bioinformatics (http://www.ilifu.ac.za/il/home; accessed on 1 October 2021).

Reverse vaccinology is a way of using bioinformatics tools to identify features in bacteria, viruses, parasites, cancer cells, or allergens that could trigger an immune response [43]. Reverse vaccinology starts with the pathogen’s genome and uses it to predict epitopes (antigens) that are most likely to be vaccine candidates. This method decreases the number of proteins to be analyzed, making the selection process easier. Through this process, one can identify antigens present in minute amounts or expressed only at specific phases, and it allows for the study of non-cultivable or dangerous microbes [44]. Immuno-informatics, a sub-discipline of bioinformatics concerned with the computational analysis of immunological data, is also a potent computational tool for vaccine development. Immuno-informatics can minimize the time and cost of vaccine development by predicting optimal antigens, epitopes, carriers, and adjuvants for vaccination. Vaccines against the Ebola virus, HIV-1, Herpes Simplex Virus (HSV)-1 and 2, human norovirus, and *Staphylococcus aureus* are examples that are under development and are using an immuno-informatics approach [45].

Overall, whole-genome sequencing projects and the rise of bioinformatics have triggered the birth of a new era of vaccine research and development, leading to a new generation of vaccines designed by deciphering the information provided by genome sequences and using it to better understand the host–pathogen interactions [46]. Riding on the capacity developed, as well as the gains from the use of bioinformatics to develop insights into the dynamics of other infectious diseases, means that South African scientists are poised to continue applying -omics informatics approaches to advance the prevention and treatment of diseases, including COVID-19 [46,47].

## 5. Conclusions and Future Perspectives

The emergence of the COVID-19 pandemic was accompanied by a global panic that resulted in an unprecedented shut down of nearly all activities, including most RDI, around the world. With nations having had time to catch their breath, attention is now being turned to how to meet the challenge posed by the novel coronavirus. South Africa is one of only a few African countries that has made significant investments in capabilities that offer leverage to combat COVID-19 and other emerging health threats. As the country regroups from the initial chaos of the pandemic, we have highlighted in this perspective piece the technological and academic resources available to the country from years of RDI investments. Leveraging these capabilities could also light a path out of the enormous economic toll the total and partial lockdowns have had on the country and the cost of procuring vaccines for the entire population. The expertise and resources exist in drug development and vaccine manufacturing: biotechnology, nanotechnology, and bioinformatics are all necessary components for a nanotechnology-based vaccine against COVID-19. Focused collaborations between scientists working in these fields—together with clinicians—are required to drive rapid, cost-effective RDI of vaccines for COVID-19. Such teams could be assembled with the support and funding from the government to research, test, and manufacture these vaccines in a local version of the US government’s Operation Warp Speed [48]. Local production has been found to be critical in ensuring adequate supplies within countries. It is without a doubt that African countries should have the capability to manufacture their own vaccines. In April 2021, the Africa Centers for Disease Control launched the Partnerships for African Vaccine Manufacturing (PAVM), having also recognized the need for local manufacturing [49]. Leveraging the vast experience in antiviral research and vaccine development for diseases, such as HIV/AIDS—together with the expertise in nanoparticle synthesis and characterization and bioinformatics—could lead to the rapid development of new vaccines and therapies. Medicines’ regulatory support during the vaccine development process is also recognized as a critical component. The capacity to review and grant marketing authorization for vaccines has been demonstrated; however, continued capacity building in this area is required [50]. The soon-to-be-established African Medicines Agency may also play a role in supporting the review of new vaccines and authorization at a continental level.

## Data Availability

Not applicable.

## References

[1] World Health Organization (2021). WHO Coronavirus (COVID-19) Dashboard. https://covid19.who.int/.

[2] Department Health Republic of South Africa (2021). COVID-19 Online Resource & News Portal SAcoronavirus.co.za. https://sacoronavirus.co.za/2021/09/13/update-on-covid-19-monday-13-september/.

[3] Cui J., Li F., Shi Z.-L. (2019). Origin and evolution of pathogenic coronaviruses. Nat. Rev. Microbiol..

[4] Gupta A., Madhavan M.V., Sehgal K., Nair N., Mahajan S., Sehrawat T.S., Bikdeli B., Ahluwalia N., Ausiello J.C., Wan E.Y. (2020). Extrapulmonary manifestations of COVID-19. Nat. Med..

[5] Elezkurtaj S., Greuel S., Ihlow J., Michaelis E.G., Bischoff P., Kunze C.A., Sinn B.V., Gerhold M., Hauptmann K., Ingold-Heppner B. (2021). Causes of death and comorbidities in hospitalized patients with COVID-19. Sci. Rep..

[6] Costanzo M., de Giglio M.A.R., Roviello G.N. (2021). Anti-Coronavirus Vaccines: Past Investigations on SARS-CoV-1 and MERS-CoV, the Approved Vaccines from BioNTech/Pfizer, Moderna, Oxford/AstraZeneca and others under Development against SARS-CoV-2 Infection. Curr. Med. Chem..

[7] Tregoning J.S., Flight K.E., Higham S.L., Wang Z., Pierce B.F. (2021). Progress of the COVID-19 vaccine effort: Viruses, vaccines and variants versus efficacy, effectiveness and escape. Nat. Rev. Immunol..

[8] Swingle K.L., Hamilton A.G., Mitchell M.J. (2021). Lipid Nanoparticle-Mediated Delivery of mRNA Therapeutics and Vaccines. Trends Mol. Med..

[9] Li Y., Tenchov R., Smoot J., Liu C., Watkins S., Zhou Q. (2021). A Comprehensive Review of the Global Efforts on COVID-19 Vaccine Development. ACS Cent. Sci..

[10] Shin M.D., Shukla S., Chung Y.H., Beiss V., Chan S.K., Ortega-Rivera O.A., Wirth D.M., Chen A., Sack M., Pokorski J.K. (2020). COVID-19 vaccine development and a potential nanomaterial path forward. Nat. Nanotechnol..

[11] Khurana A., Allawadhi P., Khurana I., Allwadhi S., Weiskirchen R., Banothu A.K., Chhabra D., Joshi K., Bharani K.K. (2021). Role of nanotechnology behind the success of mRNA vaccines for COVID-19. Nano Today.

[12] Buschmann M., Carrasco M., Alishetty S., Paige M., Alameh M., Weissman D. (2021). Nanomaterial Delivery Systems for mRNA Vaccines. Vaccines.

[13] Chaudhary N., Weissman D., Whitehead K.A. (2021). mRNA vaccines for infectious diseases: Principles, delivery and clinical translation. Nat. Rev. Drug Discov..

[14] Hou X., Zaks T., Langer R., Dong Y. (2021). Lipid nanoparticles for mRNA delivery. Nat. Rev. Mater..

[15] Rybicki E., Hitzeroth I., Meyers A., Santos M., Wigdorovitz A. (2013). Developing country applications of molecular farming: Case studies in South Africa and Argentina. Curr. Pharm. Des..

[16] Boshra H., Truong T., Nfon C., Bowden T.R., Gerdts V., Tikoo S., Babiuk L.A., Kara P., Mather A., Wallace D.B. (2015). A lumpy skin disease virus deficient of an IL-10 gene homologue provides protective immunity against virulent capripoxvirus challenge in sheep and goats. Antivir. Res..

[17] Anselmo A.C., Mitragotri S. (2021). Nanoparticles in the clinic: An update post COVID-19 vaccines. Bioeng. Transl. Med..

[18] Bobo D., Robinson K.J., Islam J., Thurecht K.J., Corrie S.R. (2016). Nanoparticle-Based Medicines: A Review of FDA-Approved Materials and Clinical Trials to Date. Pharm. Res..

[19] Batty C.J., Bachelder E.M., Ainslie K.M. (2021). Historical Perspective of Clinical Nano and Microparticle Formulations for Delivery of Therapeutics. Trends Mol. Med..

[20] Tanne J.H. (2021). COVID-19: FDA approves Pfizer-BioNTech vaccine in record time. BMJ.

[21] Dube A., Semete-Makokotlela B., Ramalapa B.E., Reynolds J., Boury F. (2021). Nanomedicines for the Treatment of Infectious Diseases: Formulation, Delivery and Commercialization Aspects.

[22] Chang E.H., Harford J.B., Eaton M.A., Boisseau P.M., Dube A., Hayeshi R., Swai H., Lee D.S. (2015). Nanomedicine: Past, present and future—A global perspective. Biochem. Biophys. Res. Commun..

[23] Dube A. (2019). Nanomedicines for Infectious Diseases. Pharm Res..

[24] Zhou D.T., Maponga C.C., Madhombiro M., Dube A., Mano R., Nyamhunga A., Machingura I., Manasa J., Hakim J., Chirenje Z.M. (2019). Mentored postdoctoral training in Zimbabwe: A report on a successful collaborative effort. J. Public Health Afr..

[25] Masara B., van der Poll J.A., Maaza M. (2021). A nanotechnology-foresight perspective of South Africa. J. Nanopart. Res..

[26] Doria-Rose N.A., Bhiman J.N., Roark R.S., Schramm C.A., Gorman J., Chuang G.-Y., Pancera M., Cale E.M., Ernandes M.J., Louder M.K. (2015). New Member of the V1V2-Directed CAP256-VRC26 Lineage That Shows Increased Breadth and Exceptional Potency. J. Virol..

[27] Mansoor L.E., Karim Q.A., Yende-Zuma N., MacQueen K.M., Baxter C., Madlala B.T., Grobler A., Karim S.S.A. (2014). Adherence in the CAPRISA 004 tenofovir gel microbicide trial. AIDS Behav..

[28] Tegally H., Wilkinson E., Giovanetti M., Iranzadeh A., Fonseca V., Giandhari J., Doolabh D., Pillay S., San E.J., Msomi N. (2021). Detection of a SARS-CoV-2 variant of concern in South Africa. Nature.

[29] Schoeman D., Cloete R., Fielding B.C. (2021). Comparative studies of the seven human coronavirus envelope proteins using topology prediction and molecular modelling to understand their pathogenicity. bioRxiv.

[30] Tshweu L.L., Shemis M.A., Abdelghany A., Gouda A., Pilcher L.A., Sibuyi N.R.S., Meyer M., Dube A., Balogun M.O. (2020). Synthesis, physicochemical characterization, toxicity and efficacy of a PEG conjugate and a hybrid PEG conjugate nanoparticle formulation of the antibiotic moxifloxacin. RSC Adv..

[31] Melariri P., Kalombo L., Nkuna P., Dube A., Hayeshi R., Ogutu B., Gibhard L., Dekock C., Smith P., Wiesner L. (2015). Oral lipid-based nanoformulation of tafenoquine enhanced bioavailability and blood stage antimalarial efficacy and led to a reduction in human red blood cell loss in mice. Int. J. Nanomed..

[32] Tshweu L., Katata L., Kalombo L., Chiappetta D.A., Hocht C., Sosnik A., Swai H. (2014). Enhanced oral bioavailability of the antiretroviral efavirenz encapsulated in poly (epsilon-caprolactone) nanoparticles by a spray-drying method. Nanomedicine.

[33] Freidus L.G., Kumar P., Marimuthu T., Pradeep P., Choonara Y.E. (2021). Theranostic Mesoporous Silica Nanoparticles Loaded with a Curcumin-Naphthoquinone Conjugate for Potential Cancer Intervention. Front. Mol. Biosci..

[34] D’Souza S., Du Plessis S., Egieyeh S., Bekale R., Maphasa R., Irabin A., Sampson S., Dube A. (2021). Physicochemical and biological evaluation of curdlan-poly(lactic-co-glycolic acid) nanoparticles as a host-directed therapy against *Mycobacterium tuberculosis*. J. Pharm. Sci..

[35] Tyavambiza C., Elbagory A., Madiehe A., Meyer M., Meyer S. (2021). The Antimicrobial and Anti-Inflammatory Effects of Silver Nanoparticles Synthesised from *Cotyledon orbiculata* Aqueous Extract. Nanomaterials.

[36] Dube P., Meyer S., Madiehe A., Meyer M. (2020). Antibacterial activity of biogenic silver and gold nanoparticles synthesized from Salvia africana-lutea and *Sutherlandia frutescens*. Nanotechnology.

[37] Kannan R., Stirk W., Van Staden J. (2013). Synthesis of silver nanoparticles using the seaweed Codium capitatum P.C. Silva (Chlorophyceae). S. Afr. J. Bot..

[38] Council on Foreign Relations (2021). South Africa’s Biovac Strikes Deal to Make COVID-19 Vaccine. https://www.cfr.org/blog/south-africas-biovac-strikes-deal-make-covid-19-vaccine.

[39] News A.H. (2021). Aspen Confirms Release of COVID-19 Vaccines to Johnson & Johnson for Supply to South Africa. https://www.aspenpharma.com/2021/07/26/aspen-confirms-release-of-covid-19-vaccines-to-johnson-johnson-for-supply-to-south-africa/.

[40] Heunis T.D.J., Botes M., Dicks L.M.T. (2010). Encapsulation of *Lactobacillus plantarum* 423 and its Bacteriocin in Nanofibers. Probiotics Antimicrob. Proteins.

[41] Mulder N.J., Christoffels A., De Oliveira T., Gamieldien J., Hazelhurst S., Joubert F., Kumuthini J., Pillay C.S., Snoep J.L., Bishop O.T. (2016). The Development of Computational Biology in South Africa: Successes Achieved and Lessons Learnt. PLoS Comput. Biol..

[42] Allam M., Ismail A., Khumalo Z.T.H., Kwenda S., van Heusden P., Cloete R., Wibmer C.K., Mtshali P.S., Mnyameni F., Mohale T. (2020). Genome Sequencing of a Severe Acute Respiratory Syndrome Coronavirus 2 Isolate Obtained from a South African Patient with Coronavirus Disease 2019. Microbiol. Resour. Announc..

[43] Enayatkhani M., Hasaniazad M., Faezi S., Gouklani H., Davoodian P., Ahmadi N., Einakian M.A., Karmostaji A., Ahmadi K. (2021). Reverse vaccinology approach to design a novel multi-epitope vaccine candidate against COVID-19: An in silico study. J. Biomol. Struct. Dyn..

[44] Soltan M.A., Magdy D., Solyman S.M., Hanora A. (2020). Design of Staphylococcus aureus New Vaccine Candidates with B and T Cell Epitope Mapping, Reverse Vaccinology, and Immunoinformatics. Omics.

[45] Oli A.N., Obialor W.O., Ifeanyichukwu M.O., Odimegwu D.C., Okoyeh J.N., Emechebe G.O., Adejumo S.A., Ibeanu G.C. (2020). Immunoinformatics and Vaccine Development: An Overview. ImmunoTargets Ther..

[46] Egieyeh S., Egieyeh E., Malan S., Christofells A., Fielding B. (2021). Computational drug repurposing strategy predicted peptide-based drugs that can potentially inhibit the interaction of SARS-CoV-2 spike protein with its target (humanACE2). PLoS ONE.

[47] Chukwudozie O.S., Gray C.M., Fagbayi T.A., Chukwuanukwu R.C., Oyebanji V.O., Bankole T.T., Adewole R.A., Daniel E.M. (2021). Immuno-informatics design of a multimeric epitope peptide based vaccine targeting SARS-CoV-2 spike glycoprotein. PLoS ONE.

[48] Kim J.H., Hotez P., Batista C., Ergonul O., Figueroa J.P., Gilbert S., Gursel M., Hassanain M., Kang G., Lall B. (2021). Operation Warp Speed: Implications for global vaccine security. Lancet Glob. Health.

[49] Irwin A. (2021). How COVID spurred Africa to plot a vaccines revolution. Nature.

[50] Semete-Makokotlela B., Mahlangu G.N., Mukanga D., Darko D.M., Stonier P., Gwaza L., Nkambule P., Matsoso P., Lehnert R., Rosenkranz B. (2021). Needs-driven talent and competency development for the next generation of regulatory scientists in Africa. Br. J. Clin. Pharmacol..

